# Diagnostic accuracy of ultrasound in evaluation of obstructive jaundice with MRCP as gold standard

**DOI:** 10.12669/pjms.36.4.1665

**Published:** 2020

**Authors:** Hina Hanif, Sohail Ahmed Khan, Sobia Muneer, Syed Omair Adil

**Affiliations:** 1 Hina Hanif, Resident, Dow Institute of Radiology, Dow University of Health Sciences, Karachi, Pakistan; 2 Sohail Ahmed Khan, Assistant Professor, Dow Institute of Radiology, Dow University of Health Sciences, Karachi, Pakistan; 3 Sobia Muneer, Resident, Dow Institute of Radiology, Dow University of Health Sciences, Karachi, Pakistan; 4 Syed Omair Adil, Lecturer, School of Public Health, Dow University of Health Sciences, Karachi, Pakistan

**Keywords:** Choledocholithiasis, MRCP, Obstructive jaundice, Sensitivity, Ultrasound

## Abstract

**Objective:**

To evaluate the diagnostic accuracy of ultrasound in obstructive jaundice taking MRCP as gold standard.

**Methods:**

This cross-sectional study was conducted at Dow Institute of Radiology (DIR), Dow University of Health Sciences (DUHS), Karachi from 2^nd^ May 2018 till 2^nd^ November 2018. Both male and female patients aged 30 to 80 years with suspected obstructive jaundice were included. Patients already diagnosed with obstructive jaundice were excluded. MRCP and ultrasound were performed in suspected patients. Diagnostic accuracy including sensitivity, specificity, positive predictive value (PPV) and negative predictive value (NPV) of obstructive jaundice were calculated using contingency tables using MRCP findings as gold standard.

**Results:**

Mean age of the patients was 54.73 ± 12.65 years. In causes of obstruction, choledocholothiasis was responsible for 85 (35.1%), stricture 61 (25.2%), carcinoma of head of pancreas 39 (16.1%), periampullary carcinoma 21 (8.7%), cholangiocarcinoma 10 (4.1%) and gallbladder carcinoma 26 (10.7%) of the cases. Diagnostic accuracy of ultrasound in obstructive jaundice taking MRCP findings as gold standard showed sensitivity, specificity, positive predicted value (PPV), negative predicted value (NPV) and overall diagnostic accuracy as 84.57%, 79.10%, 91.36%, 66.25% and 83.06%.

**Conclusion:**

Ultrasound has a high sensitivity, moderate specificity, and high diagnostic accuracy in diagnosis of obstructive jaundice.

## INTRODUCTION

Obstructive jaundice is a common surgical problem that occurs when there is an obstruction to the passage of conjugated bilirubin from liver cells to intestine.^1^ Jaundice and pain are the most common presenting complaints in patients with hepatobiliary disease. Acute biliary tract diseases cause significant morbidity and mortality, and about 2% of all admissions to hospital are for hepatobiliary diseases. Acute pancreatitis (0.54% of all admissions) and acute cholecystitis (0.48%) are the leading indications for hospitalization. MRCP is an important noninvasive imaging investigation in the preoperative evaluation of patients with obstructive jaundice.^2^-^4^ It plays a primary role in the workup and therapeutic operative planning of obstructive jaundice.^5^

Ultrasound has easy accessibility, speed, ease of performance and low cost.^6^ The diagnostic accuracy of U/S in differentiating obstructive from non-obstructive jaundice is estimated to be high in the order of about 90%.^1^,^7^ MRCP has a very high sensitivity and specificity of 98% each in diagnosis of obstructive jaundice as compared to ultrasound which has sensitivity and specificity of 88% each respectively.^2^ According to a study 17.1% of cases of jaundice are due to obstruction commonly with involvement of pancreatic and biliary system.^8^

Obstruction of pancreatobiliary system is responsible for development of obstructive jaundice. It is a common clinical problem and it is important to evaluate it quickly for prompt treatment and prevent complications. To the best of our knowledge and after going through various search engines, scarce data was retrieved on the diagnostic accuracy of ultrasonography in determination of suspected cases of biliary obstruction. Many previous studies have compared the diagnostic accuracy of MRCP with ERCP in obstructive jaundice. This study evaluated the diagnostic accuracy of ultrasound in diagnosis of obstructive jaundice taking MRCP findings as gold standard.

## METHODS

This cross-sectional study was conducted at Dow Institute of Radiology (DIR), Dow University of Health Sciences (DUHS), Karachi from 2^nd^ May 2018 till 2^nd^ November 2018. Approval from the Research Evaluation Unit of College of Physicians and Surgeons of Pakistan was obtained prior to conducting study (Ref #: CPSP/REU/RAD-2015-256-2108).

All patients aged 30 to 80 years of either gender suspected of obstructive jaundice referred to DIR for MRCP were consecutively enrolled. While those patients already diagnosed as obstructive jaundice and came for follow-up, pregnant women and all patients who were claustrophobic to MRI were excluded.

Sample size was calculated using sensitivity of ultrasound: 88%^2^, specificity of ultrasound: 88%^2^, obstructive jaundice prevalence: 17.1%^9^, confidence interval: 95%, and margin of error: 10%. The final sample size came out to be 242 patients. Obstructive jaundice was defined as presence of the all or any one of the following characteristics for the duration of 2 or more weeks; yellowish color of skin, sclera (yellowish coloration of eyes), and raised bilirubin level of 2–2.5 mg/dl. On ultrasound, obstructive jaundice was labeled as positive in the presence of dilatation of intra hepatic biliary duct of 2 mm or more or extrahepatic biliary duct of 4 mm or more. On MRCP, it was labeled as positive in the presence of biliary duct dilatation, intrahepatic biliary ducts 2 mm or more and extrahepatic bile ducts 6 mm or more.

On ultrasound choledocolithiasis was labeled as an echogenic focus in the common bile duct with posterior acoustic shadowing On MRCP choledocolithiasis was labeled as a hypointense filling defect in the common bile duct whereas on ultrasound stricture in the common bile duct was defined as focal segmental narrowing of lumen of common bile duct. On MRCP stricture in the common bile duct was defined as focal segmental narrowing of lumen of common bile duct anywhere along its path.

On ultrasound, carcinoma of head of pancreas was defined as a hypoechoic lesion of any size in the head of pancreas. On MRCP carcinoma of head of pancreas was defined as a heterogeneous signal intensity lesion of any size in head of pancreas. On ultrasound periampullary carcinoma was defined as a presence of a hypoechoic lesion of any size in the periampullary region. On MRCP periampullary carcinoma was defined as a heterogeneous signal intensity lesion of any size in the periampullary region.

On ultrasound cholangiocarcinoma was defined as a presence of a heterogeneous echotexture lesion of any size in the biliary system. On MRCP cholangiocarcinoma was defined as a heterogeneous signal intensity lesion of any size in the biliary system. On ultrasound gall bladder carcinoma was defined as a presence of a heterogeneous echotexture lesion of any size arising from gall bladder

On ultrasound gall bladder carcinoma was defined as a presence of a heterogeneous signal intensity lesion of any size arising from gall bladder.

Radiologists were blinded to sonography results and evaluated the MRCP and described the finding indicating the location and cause of obstruction in obstructive jaundice. While the patient was waiting to undergo MRCP, the patient underwent directly under a limited sonographic scan covering the area of pancreatobiliary region. This examination on ultrasound was performed on GE Voluson S6 machine by a trained sonographer having 3 years of experience using a curved low frequency probe (2-5 MHz). The pancreatobiliary region was evaluated with gray scale ultrasound and the sonographer was blinded to the MRCP result and described the location and cause of obstruction. This information along with demographic information like age, gender, duration of symptoms, cause of obstruction and socioeconomic status of the patients was entered in the proforma attached.

Diagnostic accuracy was evaluated by calculating sensitivity, specificity, positive and negative predictive values. Presence of obstructive jaundice detected by both ultrasound and MRCP was labeled as true positive. Presence of obstructive jaundice detected only by ultrasound and not by MRCP labeled as false positive. No obstructive jaundice detected by both ultrasound and MRCP was labeled as true negative. Presence of obstructive jaundice detected by MRCP and not by ultrasound was labeled as false negative.

Data were analyzed using SPSS version 22.0. Quantitative outcome variables such as age, and duration of symptoms were mentioned as mean and standard deviation. Qualitative outcome variables such as gender, obesity, cause of obstruction and socioeconomic status were mentioned as frequency and percentage. Diagnostic accuracy including sensitivity, specificity, positive predictive value (PPV) and negative predictive value (NPV) of ultrasound in obstructive jaundice was calculated using contingency tables using MRCP findings as gold standard. Effect modifiers such as gender, age, duration of symptoms, socioeconomic status and cause of obstruction were stratified to see the effect of these on outcome variables. Post stratification diagnostic accuracy was also calculated.

## RESULTS

Of 242 patients, mean age was 54.73 ± 12.65 years. There were 70 (28.90%) patients with ≤45 years of age and 172 (71.1%) with >45 years of age. 122 (50.4%) were males and 120 (49.6%) were males. The mean duration of symptoms was 34.33 ± 16.08 days. There were 167 (69.0%) patients with ≤40 days of duration of symptoms and 75 (31.0%) patients with >40 days of duration of symptoms. Obesity was found in 138 (57.0%). In causes of obstruction, choledocholothiasis was responsible for 85 (35.1%), stricture 61 (25.2%), carcinoma of head of pancreas 39 (16.1%), periampullary carcinoma 21 (8.7%), cholangiocarcinoma 10 (4.1%) and gallbladder carcinoma 26 (10.7%) of the cases.

Positive findings for obstructive jaundice on ultrasound were observed in 162 (66.90%) of the patients while on MRCP positive obstructive jaundice findings were observed in 175 (72.30%) of the patients. The negative values of obstructive jaundice on ultrasound were 80 (33.10%). However, obstructive jaundice on MRCP were 67(27.70%).

Diagnostic accuracy of ultrasound in obstructive jaundice taking MRCP findings as gold standard showed sensitivity, specificity, PPV, NPV and overall diagnostic accuracy as 84.57%, 79.10%, 91.36%, 66.25% and 83.06% (Table-I). Stratification was done with respect to age, gender, duration of symptoms, and cause of obstruction. Results are shown in detailed in Table-II.

**Table-I:** Diagnostic accuracy of ultrasound taking MRCP as gold standard (n=242).

Ultrasound	MRCP		

	Positive	Negative	Total
Positive	148	14	162
Negative	27	53	80

Total	175	67	242

Sensitivity: 84.57%, Specificity: 79.10%, PPV: 91.36%, NPV: 66.25%, Overall diagnostic accuracy: 83.06%.

**Table II:** Baseline characteristics and diagnostic accuracy of ultrasound taking MRCP as gold standard (n=242).

	Total (n)	Sensitivity	Specificity	PPV	NPV	Overall Diagnostic Accuracy
***Age, years***						
≤45	70	86%	70%	87.76%	66.67%	81.43%
>45	172	84%	82.98%	92.92%	66.10%	83.72%
***Gender***						
Male	122	85.56%	81.25%	92.77%	66.67%	84.43%
Female	120	83.53%	77.14%	89.87%	65.85%	81.67%
***Duration of symptoms, days***						
≤40	167	84.55%	70.45%	88.89%	62.00%	80.84%
>40	75	84.62%	95.65%	97.78%	73.33%	88.00%
***Cause of obstruction***						
Choledocholithiasis	85	84.13%	68.18%	88.33%	60.00%	80.00%
Stricture	61	88.89%	93.75%	97.56%	75.00%	90.16%
Carcinoma of head of pancreas	39	78.57%	90.91%	95.65%	62.50%	82.05%
Gall bladder carcinoma	26	76.47%	66.67%	81.25%	60.00%	73.08%
Periampullary carcinoma	21	87.50%	80.00%	93.33%	66.67%	85.71%
Cholangiocarcinoma	10	100%	75%	85.71%	100%	90.00%

PPV: Positive predicted value, NPV: Negative predicted value.

## DISCUSSION

For evaluation of obstructive jaundice, it is of prime importance that the radiologist should identify the cause and the level of obstruction. Improved high resolution radiological equipment and improved imaging techniques performed by an experienced radiologist provide effective means for diagnosis the etiology of obstructive jaundice. Imaging modalities such as ultrasound, CT, MRI, direct cholangiography and invasive methods such as ERCP can help diagnose the cause of obstructive jaundice as well as identify the level of obstruction.^10^,^11^ Ultrasound has always been used as initial screening method. Many advantages of this technique are present. It is a cost effective and non-invasive modality that is available easily. Most important advantage is its lack of ionizing radiation.^12^-^14^ Ultrasound is suited well enough to identify the common hepatic duct (CHD) and the proximal part of common bile duct.^15^

The finding of the current study showed that the sensitivity of ultrasound in cases of obstructive jaundice is 84.5%. Our reported sensitivity is slightly lower than the one reported by Singh et al. According to Singh et al, ultrasound has a sensitivity of 88% in diagnosing obstructive jaundice.^2^ Moreover, our reported sensitivity is also lower than the one reported by Kani et al., that reported 97% sensitivity.^16^ This variation in sensitivity can be attributed to the fact that distal part of common bile duct is difficult to visualize on ultrasound. Moreover, intrapancreatic part as well as ampullary region can also not be visualized well on ultrasound. Another potential reason for this could be due to the body habitus of the patient. Moreover, bowel gas shadows may also obscure the details leading to difficult visualization of the common bile duct distally.^17^

Our present study demonstrated that the specificity of ultrasound in diagnosis of obstructive jaundice is 79.10%. Singh et al reported the specificity of ultrasound to be 88% in diagnosis of obstructive jaundice.^2^ Ferrari et al. demonstrated that specificity of ultrasound in obstructive jaundice is 98.2%.^18^

The most common cause of obstructive jaundice in our study was choledocholithiasis. According to Karki et al., choledocholithiasis is the most common benign cause for obstructive jaundice.^19^ Singh et al. also reported choledocholithiasis as being the most common benign etiology for obstructive jaundice.^2^ Stones in gallbladder can slip into the common bile duct resulting in choledocholithiasis. Due to cholesterol composition, ultrasound serves better to visualise cholelithiasis and choledocholithiasis.

Among the malignant causes of obstructive jaundice, our study reported that carcinoma of head of pancreas is the most common one. However, studies by Karki et al.^19^ and Singh et al.^2^ report cholangiocarcinoma being the most common malignant cause of obstructive jaundice. This variation can be due to difference in demographics. Moreover, a difference in genetics as well as environmental factors may also have a role.

### Limitations of the study

Certain limitations exist in our study. Excessive bowel gas shadows were present in some of the patients that obscured details of the pancreas, periampullary and peripancreatic region. This might have resulted in missing some of the potential causes of obstructive jaundice on ultrasound. As the ultrasound was only performed by a single observer, therefore interobserver variability could not be evaluated. Moreover, we were not able to evaluate the intraobserver variability.

Despite these limitations, we believe that our study offered an approach for diagnosis of obstructive jaundice by the help of ultrasound. The results of present study have provided the local statistics related to diagnostic accuracy of ultrasound in diagnosis of obstructive jaundice taking findings of MRCP as a gold standard.

## CONCLUSION

Ultrasound has a high sensitivity, moderate specificity and a high diagnostic accuracy in diagnosis of obstructive jaundice. It is recommended that ultrasound can be used as a screening imaging technique to identify the presence or absence of intrahepatic biliary duct dilatation thereby shortlisting the patients for MRCP examination. If the patients are not feasible to undergo MRCP examination, then other investigations such as contrast enhanced computed tomography or ERCP can be performed in such patients.

### Authors’ Contribution

**HH, SAK** conceived and designed.

**SOA** did statistical analysis & editing of manuscript.

**HH, SB,** did data collection and manuscript writing.

**HH, SAK** takes the responsibility and is accountable for all aspects of the work in ensuring that questions related to the accuracy or integrity of any part of the work are appropriately investigated and resolved.
